# Clinical Accuracy and Patient Experience Following Robotic-Assisted Full-Arch Implant Rehabilitation: A Case Series

**DOI:** 10.3390/dj14080528

**Published:** 2026-08-18

**Authors:** Baoluo Xing Gao, Francisco G. F. Tresguerres, Natalia Monasterio Sebastian, Anna Millán Raventós, Rui Xie, Joaquín Delgado Gregori, Joaquín López-Malla Matute

**Affiliations:** 1 Faculty of Dentistry, Master Degree in Oral Surgery, Implantology and Periodontology, Alfonso X el Sabio University, 28037 Madrid, Spain; bxinggao@uax.es (B.X.G.);; 2 State Key Laboratory of Oral & Maxillofacial Reconstruction and Regeneration, National Clinical Research Center for Oral Diseases, Shaanxi Key Laboratory of Stomatology, Digital Dentistry Center, School of Stomatology, The Fourth Military Medical University, Xi’an 710032, China; xierui@fmmu.edu.cn

**Keywords:** robotic-assisted implant surgery, autonomous robotics, full-arch rehabilitation, edentulous patients, implant accuracy, patient-reported outcomes, computer-assisted implant surgery

## Abstract

**Objectives:** To evaluate the clinical accuracy and patient-reported outcomes (PROMs) of robotic-assisted full-arch implant rehabilitation performed with an autonomous robotic implant system. **Materials and Methods:** Six consecutive edentulous patients requiring implant-supported full-arch rehabilitation were treated using the Yakebot task-autonomous robotic implant system following a fully digital workflow. A total of 36 Straumann Bone Level Tapered implants were placed using a flapless approach. Implant placement accuracy was assessed by comparing planned and postoperative CBCT datasets. Coronal, apical, depth, and angular deviations were automatically calculated using dedicated robotic planning software. Patient-reported outcomes were prospectively evaluated through a structured questionnaire assessing preoperative perceptions, postoperative morbidity, and overall treatment satisfaction. **Results:** All implants were successfully placed according to the robotic-assisted workflow without intraoperative complications or conversion to conventional surgery. Mean three-dimensional coronal and apical deviations were 0.45 ± 0.18 mm and 0.47 ± 0.19 mm, respectively, while mean total angular deviation was 1.30 ± 0.63°. Patients reported low preoperative anxiety (0.5 ± 0.8/10), limited postoperative pain (2.2 ± 3.5/10), swelling (1.3 ± 2.2/10), and interference with daily activities (1.3 ± 2.2/10). Overall surgical experience was highly rated (9.7 ± 0.8/10). Final satisfaction scores were exceptionally high, with all patients indicating they would undergo robotic-assisted surgery again and recommend the procedure to others. **Conclusions:** Robotic-assisted full-arch implant rehabilitation demonstrated a high level of clinical accuracy, with submillimetric coronal and apical deviations and low angular discrepancies. In addition, treatment was associated with low postoperative morbidity, excellent patient acceptance, and very high satisfaction. These findings support the potential of autonomous robotic implant surgery as a predictable and patient-centered approach for full-arch rehabilitation, although larger controlled studies are required to confirm these results.

## 1. Introduction

Edentulism remains a significant global oral health challenge and continues to affect millions of individuals worldwide despite substantial advances in preventive and restorative dentistry. Complete tooth loss is associated with impaired masticatory efficiency, compromised phonetics, reduced nutritional intake, esthetic concerns, social limitations, and a marked deterioration in oral health-related quality of life [1]. Implant-supported full-arch rehabilitation has become a predictable and well-documented treatment modality for edentulous patients, providing high implant survival rates, improved function, enhanced patient satisfaction, and favorable long-term prosthetic outcomes [2,3]. Nevertheless, the long-term success of implant-supported full-arch prostheses depends not only on osseointegration but also on accurate prosthetically driven implant positioning.

The increasing adoption of digital technologies has profoundly transformed implant dentistry over the last two decades. The integration of cone-beam computed tomography (CBCT), intraoral scanning, virtual treatment planning, and computer-assisted implant surgery (CAIS) has facilitated a prosthetically driven approach in which implant positions are determined according to restorative requirements while respecting patient-specific anatomical limitations.

Static computer-assisted implant surgery remains the most widely adopted form of digital implant guidance. However, despite these advantages, static guidance remains susceptible to cumulative inaccuracies arising throughout the digital workflow. These limitations may be particularly relevant in edentulous patients where mucosa-supported templates rely on resilient soft tissues for stabilization and may experience displacement during surgery [4].

Dynamic navigation systems were introduced to address some of these limitations. Nevertheless, navigation systems remain highly dependent on operator skill because no physical constraint exists between the planned and executed trajectory.

Robotic computer-assisted implant surgery (r-CAIS) has emerged as the most recent evolution of digital implantology. Robotic systems integrate CBCT imaging, intraoral scanning, optical tracking technologies, stereoscopic visual positioning, robotic arm guidance, navigation systems, and real-time spatial monitoring into a unified digital workflow.

The conceptual basis of robotic surgery originates from the broader framework of human–robot collaboration. In this model, clinical decision-making remains under the responsibility of the surgeon, while robotic technologies contribute enhanced precision, reproducibility, and positional control during task execution.

Among currently available robotic platforms, the Yakebot autonomous robotic implant system represents the first robotic system specifically developed for dental implant surgery. The platform combines dedicated planning software (DentalNavi 4.0.0, Yakebot Technology Co., Beijing, China), optical tracking devices, stereoscopic cameras, robotic arm control, calibration modules, real-time navigation, and postoperative precision analysis within a single workflow.

The evidence supporting robotic implant placement has expanded considerably in recent years. Wang et al. [5] reported significantly lower coronal, apical, depth, and angular deviations compared with fully guided static surgery. Wu et al. [6] reported pooled platform, apical, and angular deviations of 0.68 mm, 0.67 mm, and 1.69°, respectively. Yoo et al. [7] further concluded that robotic implant surgery improved implant positioning accuracy compared with freehand surgery and demonstrated advantages over static CAIS.

Despite these encouraging findings, important limitations remain within the existing literature. Most available studies have focused on single implants, partially edentulous patients, experimental models, or mixed clinical populations. Furthermore, most robotic implant studies have focused almost exclusively on radiographic accuracy outcomes, whereas patient-reported outcome measures (PROMs) remain largely unexplored.

Therefore, the aim of the present prospective case series was to evaluate the clinical application of robotic-assisted full-arch implant rehabilitation using the Yakebot autonomous robotic implant system in completely edentulous patients. The primary objective was to assess implant placement accuracy through coronal, apical, depth, and angular deviations. The secondary objective was to evaluate patient perceptions regarding robotic-assisted implant surgery, including anxiety, confidence in robotic technology, perceived surgical precision, postoperative recovery, treatment acceptance, and overall satisfaction.

## 2. Materials and Methods

### 2.1. Study Design and Patient Selection

This prospective case series evaluated the clinical accuracy and patient experience associated with robotic-assisted full-arch implant rehabilitation using the Yakebot autonomous robotic implant system (Yakebot Technology Co., Beijing, China). Six consecutive completely edentulous patients requiring implant-supported full-arch rehabilitation were included and treated according to a standardized digital workflow. A total of 36 implants were placed.

All patients were informed about the nature of the treatment and provided written informed consent before participation. The study was conducted in accordance with the principles of the Declaration of Helsinki. As this was an observational case series evaluating a CE-marked robotic implant system used within its intended clinical indication, and no experimental intervention or allocation to different treatment groups was performed, formal ethics committee approval and prospective clinical trial registration were not required according to institutional regulations.

Patients presenting complete edentulism of the maxilla, mandible, or both arches and requiring implant-supported fixed full-arch rehabilitation and presenting with sufficient alveolar bone volume to allow implant placement without the need for bone augmentation procedures were considered eligible for inclusion. In addition, patients were required to have adequate soft tissue quality and quantity, including a sufficient band of keratinized mucosa, to permit a flapless surgical approach. Exclusion criteria included uncontrolled systemic disease, active oral infection, pregnancy, previous head and neck radiotherapy, intravenous antiresorptive therapy, severe immunosuppression, or any medical condition contraindicating implant surgery.

### 2.2. Digital Data Acquisition and Prosthetically Driven Planning

The digital workflow began with acquisition of radiographic and surface data.

A cone-beam computed tomography (CBCT) scan was obtained for each patient using a Carestream CS 9300 (Carestream Dental, Atlanta, GA, USA) unit with a voxel size of 0.2 mm. During image acquisition, patients wore a radiographic prosthetic template incorporating radiopaque fiducial markers. Surface data were acquired using a TRIOS 3 intraoral scanner (3Shape, Copenhagen, Denmark).

The CBCT-derived DICOM datasets and STL files were imported into DentalNavi version 4.0.0 (Yakebot Technology Co., Beijing, China) Registration was performed using landmark-based registration followed by point-cloud refinement and visual verification.

Virtual implant planning was performed according to prosthetically driven principles. All implants corresponded to the Straumann Bone Level Tapered (BLT) implant system (Institut Straumann AG, Basel, Switzerland).

Following completion of virtual planning, a customized guide was designed and manufactured to facilitate placement of the robotic registration pins.

### 2.3. Anchor-Pin Registration Protocol

Using the customized guide, three anchor pins were inserted into the alveolar bone before surgery. The anchor pins were positioned in a broad triangular configuration and distributed across the arch to maximize three-dimensional spatial stability while avoiding interference with planned implant positions.

In addition to the three anchor pins, a fourth centrally positioned fixation pin was inserted to support the optical tracking marker used during robotic navigation.

After placement of the anchor pins and optical marker support, a second CBCT scan was obtained using the same imaging parameters as the initial scan. The second DICOM dataset was imported into DentalNavi and matched with the original virtual treatment plan, establishing the definitive spatial relationship between patient anatomy, planned implant positions, and the robotic coordinate system.

### 2.4. Robot Calibration and Registration

Before surgery, robotic calibration procedures were performed according to the manufacturer’s protocol.

The optical tracking marker attached to the central fixation pin served as the primary reference structure for real-time navigation. Calibration probes supplied by the manufacturer were used to establish the spatial mapping relationship between the robotic arm, the optical tracking system, and the CBCT-derived treatment plan.

The calibration procedure generated a unified coordinate system linking patient anatomy, surgical instruments, optical tracking devices, and virtual implant planning.

### 2.5. Robotic-Assisted Surgical Procedure

All surgeries were performed under local anesthesia using a flapless approach.

The robotic system guided osteotomy preparation according to the preoperative virtual treatment plan. During surgery, stereoscopic cameras continuously monitored the spatial relationship between the patient, the robotic arm, and the surgical instruments. The robotic arm controlled drill positioning, angulation, entry point, and depth according to the predefined implant trajectory.

Before initiating the implant osteotomy sequence, a 3.5 mm round bur from the Yakebot surgical kit was used in all cases to flatten the cortical bone at the intended implant entry point. This maneuver eliminated cortical irregularities and crestal prominences, creating a stable reference surface for subsequent drilling. The objective was to reduce the risk of drill slippage on inclined cortical surfaces and enhance the precision of osteotomy preparation.

Following cortical flattening, implant site preparation was completed according to the manufacturer’s recommended drilling protocol for the Straumann BLT implant system. Particular attention was given to the use of the profile drill, which was employed in all osteotomies. Unlike conventional freehand surgery, where drill vibration, hand instability, or minor deviations may discourage the routine use of profile drills in certain clinical situations, the high positional accuracy and stability provided by the robotic system allowed predictable execution of every drilling step as planned. Consequently, profile drilling was consistently performed to optimize crestal implant seating and ensure full adherence to the recommended surgical protocol. Finally, implants were subsequently inserted following robotic guidance.

Throughout the procedure, the surgeon remained responsible for all clinical decision-making, patient preparation, local anesthesia, anchor-pin placement, calibration verification and continuous supervision of the robotic workflow. The autonomous robotic system executed osteotomy positioning, drilling and implant insertion according to the predefined treatment plan under continuous optical tracking and surgeon supervision. Therefore, the system should be considered task-autonomous rather than fully autonomous.

### 2.6. Postoperative Imaging and Accuracy Analysis

Immediately after surgery, a third CBCT scan was acquired using the same imaging protocol employed during treatment planning (CS 9300, Carestream Dental, Atlanta, GA, USA; voxel size 0.2 mm).

Accurate matching of the three CBCT datasets requires that all scans be performed using the same CBCT unit and identical acquisition parameters. Following the ALADA principle, exposure settings were kept as low as diagnostically acceptable while maintaining sufficient image quality for accurate registration. Although three CBCT scans were required as part of the robotic registration protocol, this represents a necessary trade-off to achieve precise patient registration and postoperative accuracy assessment.

The postoperative DICOM dataset was imported into DentalNavi 4.0.0 (Yakebot, Beijing, China) and superimposed onto the original virtual treatment plan. Following image registration, the integrated DentalNavi accuracy analysis module automatically calculated discrepancies between planned and achieved implant positions.

**Figure 1:**
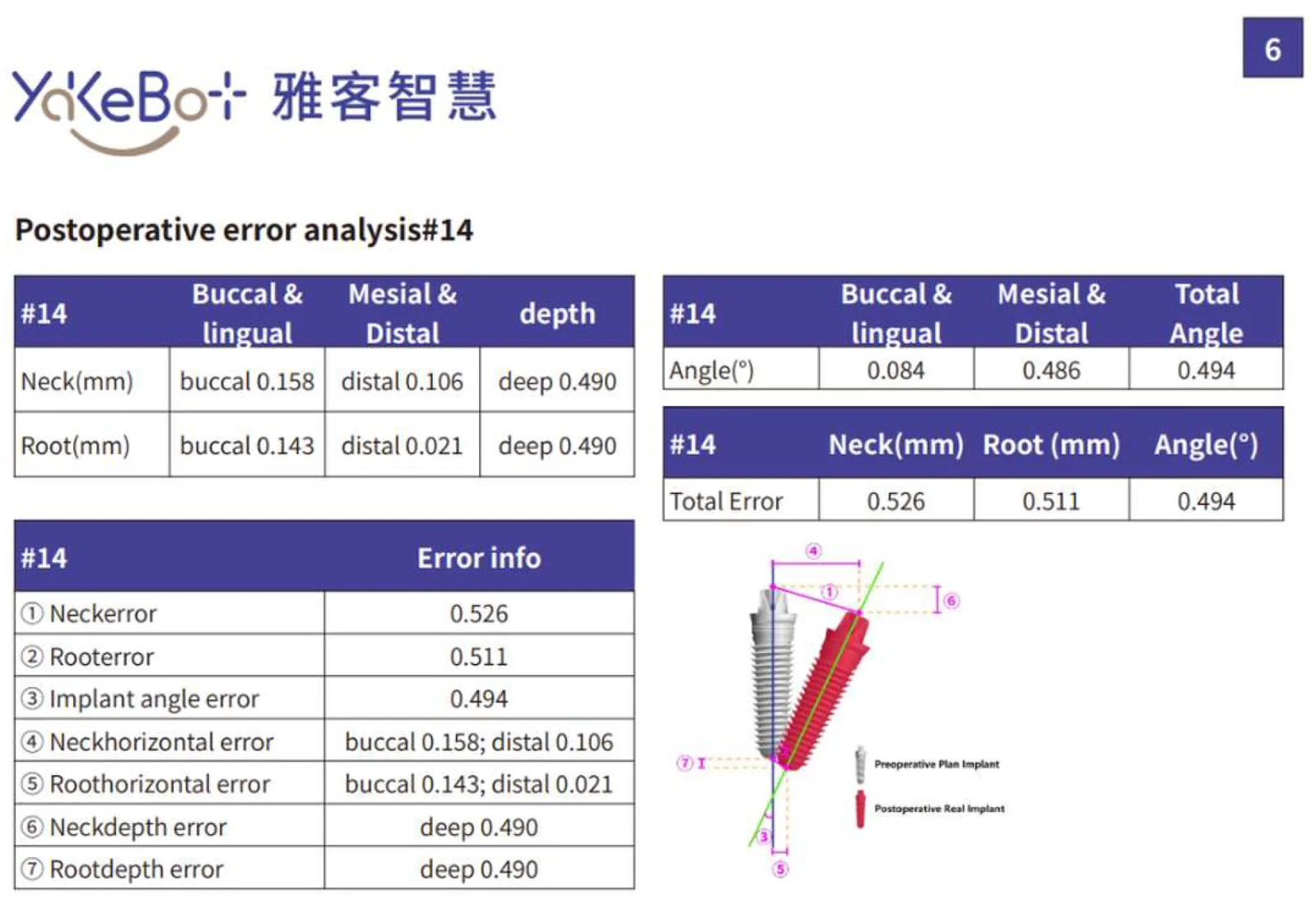
Representative postoperative accuracy report generated by the robotic navigation software. The report illustrates the methodology used to assess implant placement accuracy by comparing the planned and actual implant positions and calculating coronal (platform), apical (root), depth, and angular deviations.

Implant placement accuracy was assessed using linear and angular deviation measurements. Neck bucco-lingual, neck mesio-distal, and neck depth deviations (mm) represented the discrepancies between the planned and actual implant positions at the coronal platform level along the bucco-lingual, mesio-distal, and vertical axes, respectively. Similarly, apex bucco-lingual, apex mesio-distal, and apex depth deviations (mm) described the corresponding positional differences at the implant apex. Total coronal deviation (mm) and total apical deviation (mm) were calculated as the three-dimensional Euclidean distances between the planned and placed implant positions at the implant neck and apex, respectively. Angular accuracy was evaluated through bucco-lingual angular deviation (°), mesio-distal angular deviation (°), and total angular deviation (°), which quantified discrepancies in implant inclination within the respective planes and in three-dimensional space overall. Lower values indicated greater agreement between the planned and achieved implant positions (Figure 1).

The following outcome variables were recorded for each implant:

Neck bucco-lingual deviation (mm);

Neck mesio-distal deviation (mm);

Neck depth deviation (mm);

Apex bucco-lingual deviation (mm);

Apex mesio-distal deviation (mm);

Apex depth deviation (mm);

Total coronal deviation (mm);

Total apical deviation (mm);

Bucco-lingual angular deviation (°);

Mesio-distal angular deviation (°);

Total angular deviation (°).

Image registration and postoperative accuracy assessment were performed by an experienced investigator using the integrated DentalNavi software following the manufacturer’s standardized workflow. After image superimposition, all positional deviations were automatically calculated by the software, minimizing operator-dependent measurement variability. Because the same investigator performed the analyses and was aware of the treatment protocol, blinding was not feasible.

All measurements were automatically generated by the DentalNavi accuracy assessment software and exported for statistical analysis.

### 2.7. Patient-Reported Outcome Measures

As no validated questionnaire specifically addressing patient perceptions of robotic-assisted full-arch implant rehabilitation was available, a study-specific PROMs questionnaire was developed by the investigators. Prior to its clinical use, the questionnaire underwent expert review by clinicians experienced in implant dentistry and robotic-assisted surgery to assess its clarity, content relevance, and comprehensibility for patients. Formal psychometric validation was not performed.

PROMs were prospectively collected using a structured questionnaire specifically designed to evaluate patient perceptions of robotic-assisted implant surgery (File S1).

Preoperative questionnaires evaluated anxiety, confidence in robotic surgery, perceived benefits of robotic technology, treatment expectations, and anticipated postoperative discomfort.

Postoperative assessments were performed at 7 days after surgery. Final satisfaction questionnaires were completed between one and two months after surgery using a five-point Likert scale.

### 2.8. Statistical Analysis

Descriptive statistical analysis was performed for all study variables. Continuous data were reported as mean values, standard deviations, medians, minimum values, and maximum values. Given the exploratory design and limited sample size, no inferential statistical comparisons were performed.

Because this exploratory study included only six patients, statistical analysis was limited to descriptive statistics. Although multiple implants were placed in individual patients, no inferential analyses or multilevel statistical models accounting for clustering were performed. Therefore, implant-level measurements are presented descriptively without formal statistical comparisons.

## 3. Results

### 3.1. Implant Placement Accuracy

A total of 36 implants were placed in 6 patients and included in the accuracy analysis. All implants were successfully inserted according to the robotic-assisted workflow and no intraoperative deviations requiring conversion to conventional implant placement were recorded.

**Figure 2:**
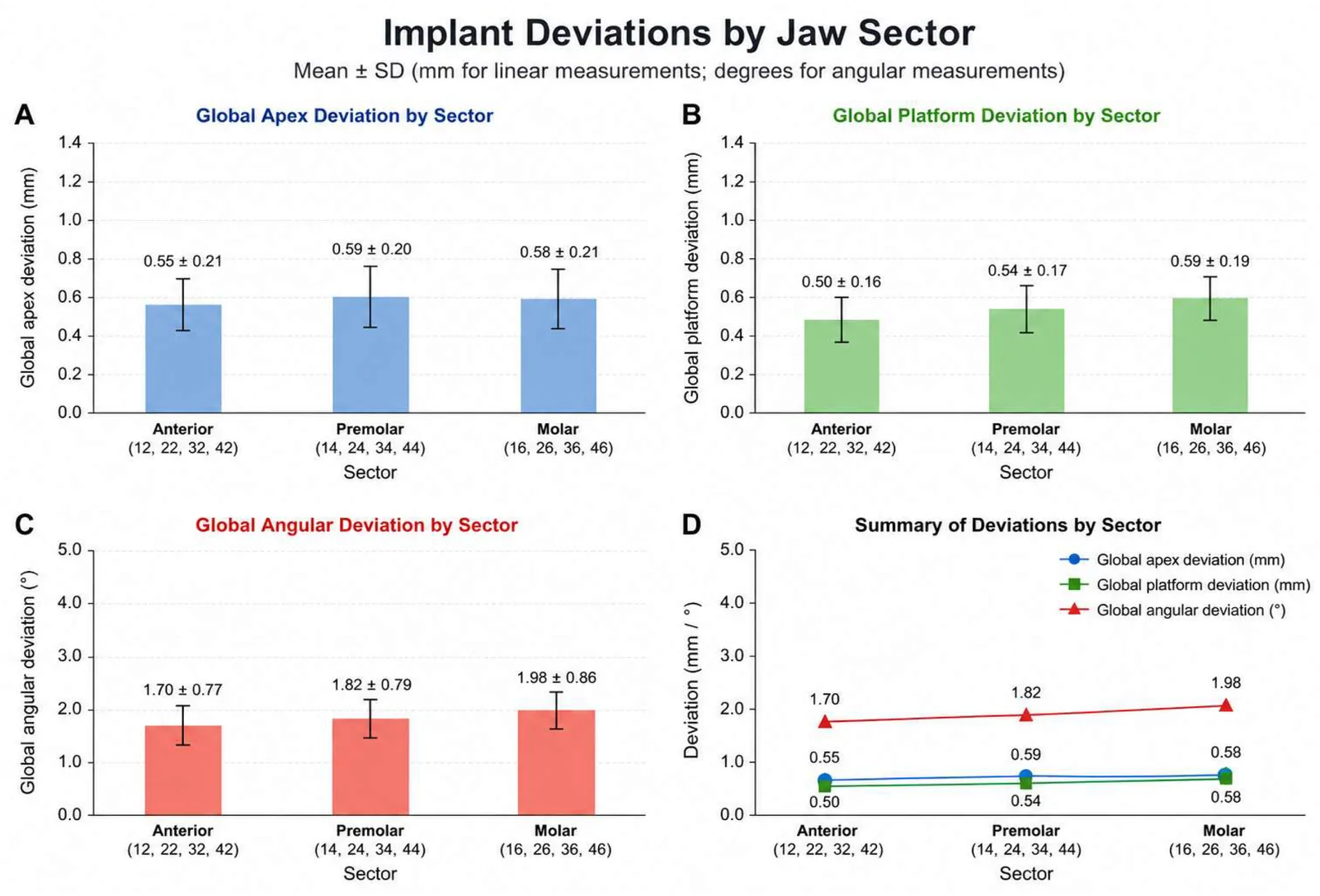
Mean ± standard deviation of global apical, platform, and angular deviations according to implant location (anterior, premolar, and molar sectors). (**A**) Global apical deviation (mm); (**B**) global platform deviation (mm); (**C**) global angular deviation (°); (**D**) comparison of global apical, platform, and angular deviations according to implant location.

Comparison between the planned and postoperative implant positions demonstrated a high level of placement accuracy. The mean three-dimensional coronal deviation was 0.45 ± 0.18 mm, while the mean three-dimensional apical deviation was 0.47 ± 0.19 mm. The mean total angular deviation was 1.30 ± 0.63° (Figure 2).

At the implant neck, mean deviations were 0.21 ± 0.16 mm in the bucco-lingual direction, 0.22 ± 0.17 mm in the mesio-distal direction, and 0.22 ± 0.19 mm in depth. At the implant apex, mean deviations were 0.26 ± 0.19 mm bucco-lingually, 0.20 ± 0.18 mm mesio-distally, and 0.22 ± 0.19 mm in depth.

Angular discrepancies measured in the bucco-lingual and mesio-distal planes were 0.83 ± 0.67° and 0.83 ± 0.51°, respectively.

Detailed accuracy measurements are presented in Table 1.

Overall, robotic-assisted implant placement demonstrated submillimetric coronal and apical deviations and angular deviations below 2°, indicating a high degree of correspondence between the planned and achieved implant positions throughout the full-arch rehabilitation procedures.

### 3.2. Patient-Reported Outcome Measures

All six patients completed the PROMs questionnaire at all evaluation time points. Preoperatively, participants reported very low levels of anxiety (0.5 ± 0.8 on a 10-point VAS) and high confidence in robotic-assisted surgery (9.3 ± 1.2). Expectations regarding both treatment outcomes (9.7 ± 0.8) and the potential of robotic technology to improve surgical accuracy (8.7 ± 1.9) were consistently high. Fear of postoperative pain remained relatively low despite the extensive nature of full-arch implant rehabilitation (2.83 ± 3.71).

At the 7-day postoperative assessment, patient-reported morbidity was limited. Mean pain scores were low (2.2 ± 3.5), while perceived swelling (1.3 ± 2.1), difficulty chewing (1.7 ± 2.6), difficulty speaking (1.7 ± 2.6), and interference with daily activities (1.3 ± 2.1) remained minimal. The need for analgesic medication was also low overall (3.50 ± 4.37). Importantly, the overall surgical experience received a highly favorable rating, with a mean score of 9.67 ± 0.82.

The final satisfaction assessment performed 1–2 months after surgery demonstrated exceptionally positive patient perceptions. Scores ranged from 4.5 to 5.0 out of 5 across all satisfaction domains. Patients considered the procedure more comfortable and less invasive than expected, reported a strong sense of safety associated with robotic assistance, and consistently perceived the treatment as highly precise. Recovery was also rated very positively (4.8 ± 0.4). Notably, all patients stated that they would choose robotic-assisted surgery again and would recommend the treatment to other patients, resulting in maximum scores for both variables (5.0 ± 0.0). Overall satisfaction with treatment was uniformly high, with all participants assigning the highest possible rating. Detailed PROMs results for the preoperative, postoperative (7 days), and final satisfaction (1–2 months) assessments are presented in Table 2.

These findings suggest that robotic-assisted full-arch implant rehabilitation is associated not only with high surgical accuracy but also with excellent patient acceptance, limited postoperative morbidity, and very high levels of satisfaction during the early healing period.

## 4. Discussion

The present prospective case series showed very high implant placement accuracy for robotic-assisted full-arch rehabilitation. Mean three-dimensional coronal and apical deviations were 0.45 ± 0.18 mm and 0.47 ± 0.19 mm, respectively, while mean total angular deviation was 1.30 ± 0.63°. To the best of our knowledge, these values represent among the lowest clinical coronal and apical deviations reported for robotic-assisted implant placement in fully edentulous or full-arch patients. This finding is particularly relevant because full-arch implant placement is usually more demanding than single-tooth or partially edentulous surgery due to the absence of stable dental references, the need for multiple implants, the importance of inter-implant distribution, and the direct prosthetic consequences of small positional deviations.

**Figure 3:**
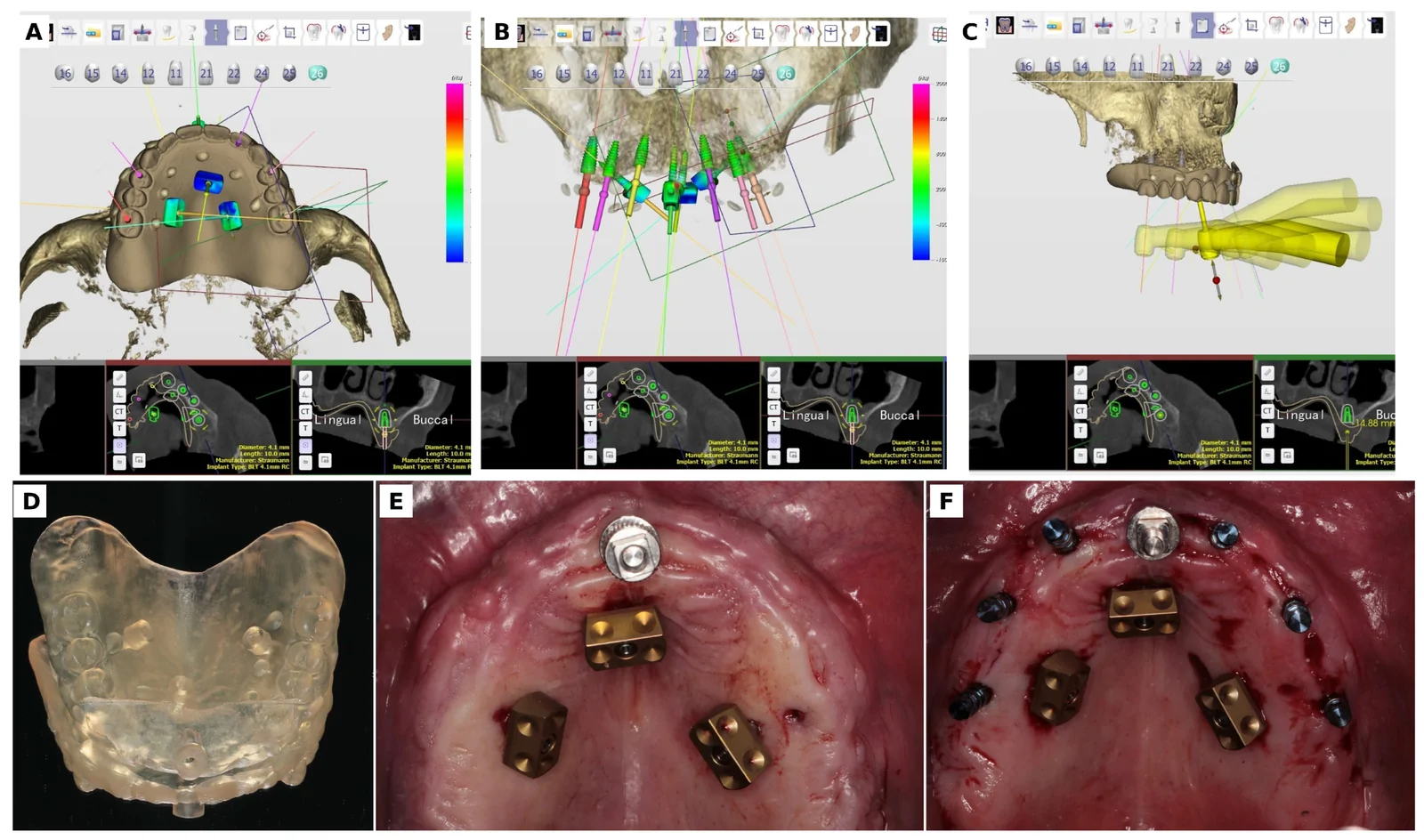
Representative workflow of robotic-assisted full-arch rehabilitation in the maxilla. (**A**) Initial virtual planning of implant and anchor-pin positions based on the preoperative CBCT and intraoral scan. (**B**) Implant planning refinement after acquisition of the second CBCT with anchor pins in place. (**C**) Virtual positioning of the robotic handpiece and drill trajectories for each implant site. (**D**) Anchor-pin surgical guide. (**E**) Intraoral placement of anchor pins and optical tracking markers prior to registration. (**F**) Final implant placement following robotic-assisted osteotomy preparation.

**Figure 4:**
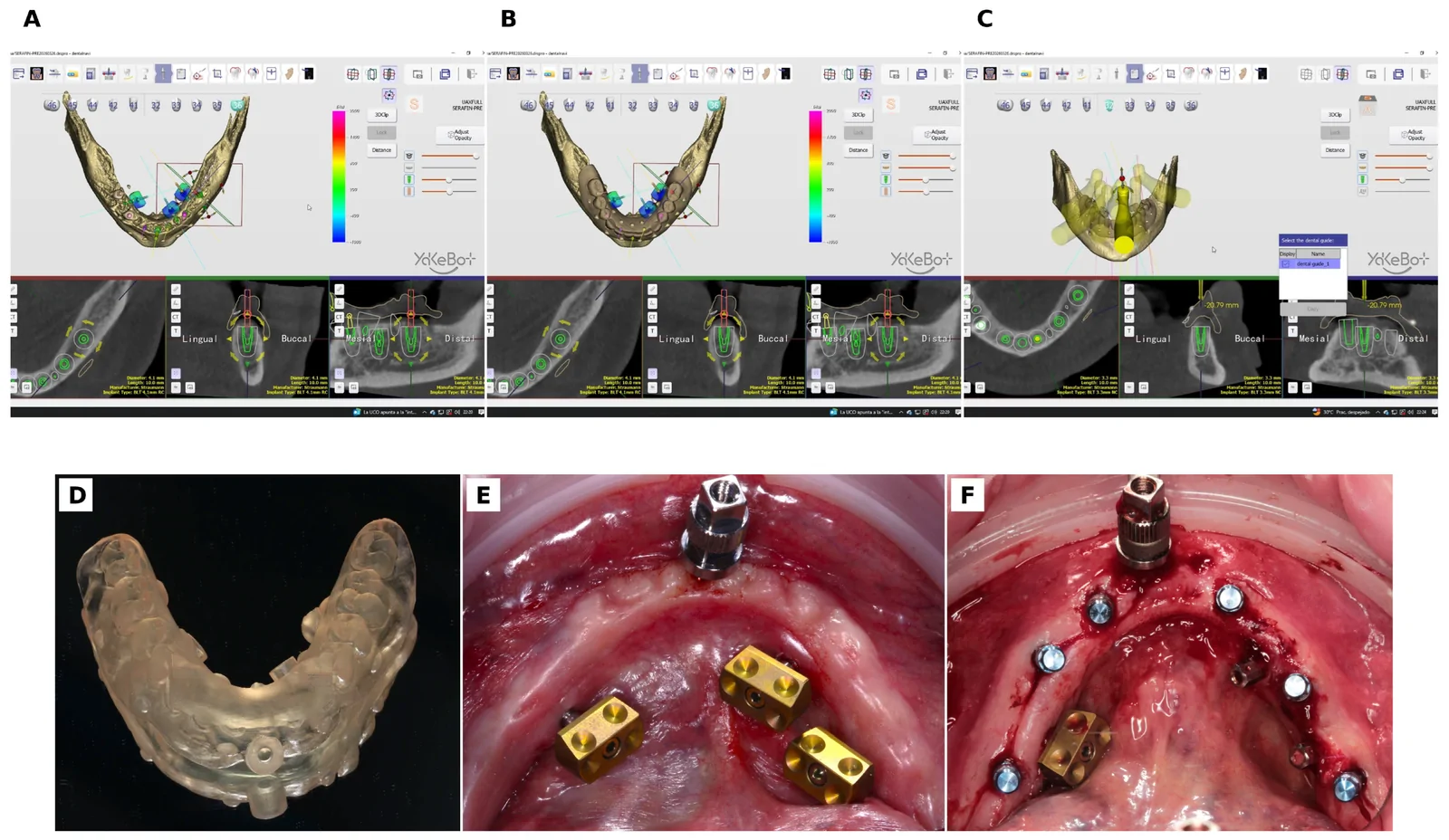
Representative workflow of robotic-assisted full-arch rehabilitation in the mandible. (**A**) Initial virtual planning of implant and anchor-pin positions based on the preoperative CBCT and intraoral scan. (**B**) Implant planning refinement after acquisition of the second CBCT with anchor pins in place. (**C**) Virtual positioning of the robotic handpiece and drill trajectories for each implant site. (**D**) Anchor-pin surgical guide. (**E**) Intraoral placement of anchor pins and optical tracking markers prior to registration. (**F**) Final implant placement following robotic-assisted osteotomy preparation.

The coronal and apical deviations observed in the present study were remarkably similar across all implant sites. This finding suggests that the osteotomy trajectory was maintained consistently throughout the entire drilling sequence, with minimal drift occurring as drilling depth increased. If substantial deviations had been introduced during osteotomy preparation, a greater discrepancy between coronal and apical measurements would be expected. Therefore, the comparable magnitude of coronal and apical deviations supports the high precision of the robotic drilling process and indicates excellent control of drill positioning along the planned implant axis. Figure 3 and Figure 4 illustrate the complete clinical and digital workflow for robotic-assisted full-arch rehabilitation in the maxilla and mandible, respectively.

While direct tactile feedback captured by the doctor’s hands is inherently reduced in robotic-assisted surgery, the surgeon continues to play a critical supervisory role. Visual assessment of the operative field and auditory feedback from the drilling process become valuable sources of information during osteotomy preparation. The characteristic sound generated by the bur as it advances through bone provides indirect confirmation of drilling progression and, together with continuous visual monitoring, enables the clinician to verify that the robotic system is performing the osteotomy according to the planned trajectory. Thus, robotic surgery should be viewed as a task-autonomous procedure, but as a highly controlled surgeon-supervised workflow.

When compared with the available literature on robotic-assisted implant surgery, the present results compare favorably with previous clinical studies. Li et al. [8] evaluated autonomous robotic computer-assisted implant surgery in fully edentulous and terminal dentition patients and reported mean global coronal, apical, and angular deviations of 0.67 ± 0.37 mm, 0.69 ± 0.37 mm, and 1.27 ± 0.59°, respectively. Although angular deviation was similar to that observed in the present study, our coronal and apical deviations were lower. Li et al. also emphasized that edentulous cases are affected by several specific sources of error, including marker design and fixation, registration accuracy, bone morphology, implant site quality, and the need for stable spatial linkage between the robotic arm and the patient. In this context, the lower linear deviations observed in the present series may be related to the standardized anchor-pin workflow, the use of repeated CBCT-based registration, and the integration of postoperative accuracy analysis within the same robotic planning environment.

Wang et al. [5] directly compared the Yakebot robotic system with fully guided static computer-assisted implant surgery in edentulous jaw implantation. In their robotic group, mean platform, apical, depth, and angular deviations were 0.65 ± 0.25 mm, 0.65 ± 0.22 mm, 0.49 ± 0.24 mm, and 1.43 ± 1.18°, respectively, whereas the fully guided static CAIS group showed significantly higher deviations of 1.37 ± 0.72 mm, 1.28 ± 0.68 mm, 0.88 ± 0.47 mm, and 3.47 ± 2.02°, respectively. Compared with that study, the present series showed lower coronal and apical deviations and a slightly lower angular deviation. This reinforces the potential value of autonomous robotic systems in full-arch surgery, especially when compared with mucosa-supported or fully guided static templates in edentulous patients.

The present results compare favorably to the pooled estimates reported in recent systematic reviews of robotic implant surgery. Wu et al. [6], in a systematic review and meta-analysis including 8 clinical studies with 109 patients and 242 implants, reported pooled clinical platform, apical, and angular deviations of 0.68 mm, 0.67 mm, and 1.69°, respectively. Similarly, Luo et al. [9] reported pooled global platform, apical, and angular deviations for robotic CAIS of 0.69 mm, 0.72 mm, and 1.62°, respectively. An umbrella review by Basualdo Allende et al. [10] reported that robotic-assisted implant surgery consistently demonstrated the highest placement accuracy among digital guidance systems, with pooled coronal deviations of approximately 0.60–0.73 mm, apical deviations of 0.63–0.70 mm, and angular deviations typically between 1.4° and 1.7°. Therefore, the coronal and apical deviations observed in the present study were below the pooled ranges reported in the most recent systematic and umbrella reviews, while angular deviation was within or slightly below the expected robotic range.

Direct comparison with other robotic systems further highlights the accuracy achieved in the present study. Yang et al. [11] evaluated a novel semi-autonomous robotic-assisted surgical system (sa-RASS) in a randomized clinical trial and reported mean global platform, apical, and angular deviations of 0.93 ± 0.50 mm, 1.07 ± 0.63 mm, and 3.10 ± 1.68°, respectively. Similarly, Shi et al. [12] reported platform, apical, and angular deviations of approximately 0.89 mm, 1.04 mm, and 2.63° using a haptic and machine-vision-controlled collaborative robotic system. In comparison, the present study demonstrated substantially lower coronal and apical deviations (0.45 ± 0.18 mm and 0.47 ± 0.19 mm, respectively), while also achieving lower angular deviation (1.30 ± 0.63°). These findings suggest that fully autonomous robotic workflows based on rigid fixation, dual-CBCT registration, and optical tracking may further reduce transfer errors compared with currently available semi-autonomous robotic platforms. When compared with haptic robotic guidance, the present results also appear favorable in terms of linear accuracy. Neugarten [13], in a large clinical series of 273 implants placed using haptic robotic guidance, reported mean coronal and apical deviations of 1.10 ± 0.69 mm and 1.12 ± 0.69 mm, respectively, with a global angular deviation of 1.42 ± 1.53°. Although that study included a substantially larger number of implants and a different robotic platform, the present series showed lower coronal and apical deviations, with a similar angular discrepancy. These differences should be interpreted cautiously because robotic systems differ in their degree of autonomy, fiducial registration strategy, tracking method, surgical indication, and operator workflow. However, they suggest that autonomous robotic workflows with rigid patient fixation may be particularly advantageous in controlling linear transfer error.

The comparison with static computer-assisted implant surgery further highlights the accuracy of the present workflow. Static CAIS has been widely validated and remains a predictable approach for prosthetically driven implant placement. Nevertheless, systematic reviews have consistently reported greater deviations than those observed in the present study. Tahmaseb et al. [14] reported mean deviations of approximately 1.2 mm at the entry point, 1.4–1.5 mm at the apex, and 3.5° angular deviation for static computer-aided implant surgery. In randomized controlled trials, Tattan et al. [15] also concluded that static computer-aided implant surgery improves accuracy compared with partially guided or freehand placement, but it remains affected by guide fabrication, guide seating, sleeve tolerance, surgical access, and the impossibility of intraoperative plan modification. These limitations are particularly relevant in edentulous patients, where mucosal resilience and guide instability may further increase deviation.

Dynamic navigation systems were developed to overcome some limitations of static guides by allowing real-time tracking, intraoperative visualization, and modification of the surgical plan. However, dynamic navigation does not physically constrain the drill trajectory, and accuracy remains dependent on the surgeon’s ability to follow the virtual target. Wei et al. [16], in a systematic review and meta-analysis, reported mean global platform, apical, and angular deviations of 1.02 mm, 1.33 mm, and 3.59°, respectively, for dynamic navigation. These values are markedly higher than those observed in the present robotic series. The most recent umbrella-level evidence also suggests that dynamic navigation may reach accuracy comparable to static CAIS when the operator is experienced, but it remains more sensitive to operator experience, tracking calibration, and visual feedback interpretation.

More recently, Wang et al. [17] performed a systematic review and meta-analysis directly comparing robotic computer-assisted implant surgery (rCAIS) with static and dynamic computer-assisted implant surgery. The authors concluded that robotic systems consistently demonstrated superior positional accuracy compared with both alternative digital workflows. Across the included studies, robotic-assisted implant placement showed significantly lower global platform, apical, and angular deviations than static and dynamic guidance approaches. These findings further support the concept that robotic systems may reduce cumulative transfer errors by combining virtual planning, real-time tracking, and mechanically controlled execution within a single workflow. The favorable accuracy values observed in the present study are consistent with the conclusions of Wang et al. [17] and further reinforce the potential advantages of autonomous robotic implant placement in demanding full-arch rehabilitations.

The results of the present study compare favorably with those reported by Xie et al. [18], who evaluated autonomous robotic-assisted full-arch implant surgery in 12 patients and 102 implants. They reported mean global coronal, apical, and angular deviations of 0.53 ± 0.19 mm, 0.58 ± 0.17 mm, and 1.83 ± 0.82°, respectively. In contrast, the present study demonstrated lower mean coronal and apical deviations (0.45 ± 0.18 mm and 0.47 ± 0.19 mm, respectively) as well as a lower angular deviation (1.30 ± 0.63°), suggesting a high level of accuracy in robotic-assisted full-arch implant placement. These differences may be related to variations in workflow, registration protocols, implant systems, or surgical execution, although direct comparisons should be interpreted cautiously given the differences in study design and sample size.

These observations are supported by the recent umbrella review by Mehta et al. [19], which synthesized evidence from eight systematic reviews and meta-analyses comprising 5130 implants. The authors concluded that robot-assisted implant surgery provides the highest positional accuracy among currently available implant placement modalities, outperforming both static and dynamic computer-assisted implant surgery in most evaluated studies. Reported robotic deviations generally ranged from 0.46 to 0.81 mm coronally, 0.50 to 1.08 mm apically, and 0.56° to 1.71° angularly, with Yakebot identified among the systems demonstrating the lowest apical deviations. Therefore, the deviations observed in the present study were below or at the lower limit of the ranges reported in the current umbrella-level evidence, further supporting the high precision achievable with autonomous robotic implant placement.

The superior linear accuracy observed in the present series may be explained by several factors. First, robotic systems combine virtual planning, real-time tracking, and mechanical or autonomous control of the drilling trajectory. Unlike dynamic navigation, the final trajectory is not only visually displayed but also physically controlled by the robotic platform. Second, the use of anchor pins in the present workflow provided a rigid reference system in fully edentulous arches, reducing one of the main sources of error associated with mucosa-supported static guides. Third, the repeated CBCT protocol allowed the virtual plan to be registered to the patient after anchor-pin placement, thereby reducing discrepancies between preoperative planning and intraoperative anatomy. Fourth, the same software ecosystem was used for planning, robotic execution, and postoperative accuracy assessment, which may reduce workflow-related transfer errors. Finally, several surgical factors may have further contributed to the high level of accuracy observed. In all cases, cortical flattening was performed using a 3.5-mm round bur prior to osteotomy preparation, creating a stable entry point and reducing the risk of drill skidding on irregular crestal surfaces. Furthermore, the profile drill was used in every implant site. The high stability of robotic drilling, characterized by minimal vibration and negligible deviation from the planned trajectory, allowed complete adherence to the manufacturer’s recommended drilling sequence. These measures may have improved osteotomy reproducibility and contributed to the remarkably low coronal and apical deviations recorded in the present study.

Interestingly, coronal and apical deviations were highly comparable throughout the study. This finding suggests that positional errors were not amplified as drilling depth increased, indicating excellent maintenance of the planned trajectory during osteotomy preparation. The close agreement between coronal and apical values supports the hypothesis that the robotic system was able to reproduce the planned implant axis with a high degree of precision, minimizing lateral drift throughout the drilling sequence.

The importance of adhering to the complete drilling protocol extends beyond mechanical accuracy. Bratu et al. [20] demonstrated that shortened drilling protocols may influence early crestal bone remodeling, supporting the biological rationale for performing the complete osteotomy sequence, including cortical flattening and profile drilling when clinically indicated. This standardized approach is particularly relevant in robotic-assisted implant surgery, where precise execution of the planned osteotomy is essential to achieve predictable clinical outcomes.

From a clinical perspective, the magnitude of deviation observed in this study may be especially relevant for full-arch rehabilitation. Small deviations in implant position can affect prosthetic emergence profile, screw-access channel location, restorative space, implant parallelism, inter-implant distribution, and passive fit of the prosthesis. In flapless full-arch surgery, where direct visualization of the alveolar bone is limited, the reliability of the digital transfer protocol becomes even more important. The submillimetric coronal and apical deviations observed in the present study therefore support the potential role of robotic-assisted surgery as a highly precise strategy for complex full-arch implant rehabilitation.

However, these findings should be interpreted cautiously. Although the present accuracy values compare very favorably with those reported for static CAIS, dynamic navigation, and other robotic systems, the sample size was limited to 6 patients and 36 implants, and no direct control group was included. Moreover, indirect comparisons with published studies are affected by differences in surgical indication, implant system, edentulism status, registration protocol, CBCT acquisition, software measurement method, robotic platform, and operator experience. Recent systematic reviews have also emphasized that most robotic implant studies remain small, non-randomized, and focused primarily on radiographic accuracy rather than long-term clinical outcomes. Therefore, although the present study suggests excellent geometric accuracy, it has several limitations. First, the sample size was small and no control group was included, limiting the ability to establish comparative conclusions. Second, the follow-up period was limited to the early postoperative phase and therefore does not allow assessment of long-term implant survival, prosthetic complications, biological outcomes, or marginal bone stability. Consequently, these findings should be regarded as preliminary and require confirmation in larger prospective controlled clinical studies with longer follow-up.

Within these limitations, the present case series demonstrates that robotic-assisted full-arch implant placement can achieve highly accurate implant positioning, with coronal and apical deviations below 0.5 mm and angular deviation close to 1°. These values are lower than most pooled estimates reported for robotic implant surgery and substantially lower than those commonly reported for static guided surgery and dynamic navigation. The findings support the potential use of autonomous robotic systems as a promising approach for complex full-arch rehabilitation and provide further evidence that robotic CAIS may represent a transition from operator-dependent guided surgery toward system-mediated, reproducible execution of prosthetically driven implant plans.

The PROMs results obtained in the present study revealed very high levels of patient acceptance and satisfaction with robotic-assisted full-arch implant rehabilitation. Patients reported minimal intraoperative discomfort and postoperative pain, together with excellent overall satisfaction scores. To the authors’ knowledge, this is the first clinical study specifically evaluating patient-reported outcome measures following robotic-assisted full-arch implant surgery. While most previous robotic implant studies have primarily focused on surgical accuracy and technical performance, the present findings suggest that the benefits of robotic-assisted surgery may extend beyond precision alone. The combination of flapless surgery, accurate implant placement, and a highly controlled digital workflow may contribute to a positive patient experience and reduced treatment burden. These findings are further supported by the high willingness of patients to undergo the procedure again and recommend it to others, highlighting the potential of robotic-assisted implant surgery as a patient-centered treatment modality. Furthermore, the PROMs questionnaire was specifically developed for this exploratory study and underwent expert review but not formal psychometric validation. Therefore, the patient-reported outcomes should be interpreted with appropriate caution.

These findings are consistent with previous reports on guided implant surgery, where low postoperative morbidity and high patient acceptance have been described. Vercruyssen et al. [21] reported favorable patient-centered outcomes following computer-guided flapless implant placement and found no significant differences between immediate and delayed loading protocols, suggesting that minimally invasive digital workflows may play a key role in enhancing the patient experience.

## 5. Conclusions

Within the limitations of this small prospective case series without a control group, robotic-assisted full-arch implant rehabilitation using the Yakebot autonomous robotic implant system demonstrated promising clinical accuracy, with mean coronal and apical deviations below 0.5 mm and a mean angular deviation of 1.30°. These values compare favorably with those reported for static computer-assisted implant surgery, dynamic navigation, and previously published robotic implant studies.

In addition to its high geometric accuracy, robotic-assisted surgery was associated with low postoperative morbidity, high patient acceptance, and excellent satisfaction outcomes. All patients reported a positive treatment experience and indicated that they would choose robotic-assisted surgery again and recommend the procedure to others. Further prospective controlled studies with larger sample sizes and long-term follow-up are required to confirm these findings and determine whether the observed accuracy translates into improved clinical, prosthetic, and patient-centered outcomes.

## Figures and Tables

**Table 1:** Accuracy outcomes of robotic-assisted implant placement (*n* = 36 implants).

Parameter	Mean ± SD
Neck B-L deviation (mm)	0.21 ± 0.16
Neck M-D deviation (mm)	0.22 ± 0.17
Neck depth deviation (mm)	0.22 ± 0.19
Root B-L deviation (mm)	0.26 ± 0.19
Root M-D deviation (mm)	0.20 ± 0.18
Root depth deviation (mm)	0.22 ± 0.19
Total coronal deviation (mm)	0.45 ± 0.18
Total apical deviation (mm)	0.47 ± 0.19
Angular deviation B-L (°)	0.83 ± 0.67
Angular deviation M-D (°)	0.83 ± 0.51
Total angular deviation (°)	1.30 ± 0.63

**Table 2:** Patient-reported outcome measures (PROMs).

**Preoperative Assessment (10-point VAS)**	
**Parameter**	Mean ± SD
Anxiety	0.50 ± 0.84
Confidence in robotic surgery	9.33 ± 1.21
Expected improvement in surgical accuracy	8.67 ± 1.97
Expectations regarding treatment outcome	9.67 ± 0.82
Fear of postoperative pain	2.83 ± 3.71
**Postoperative assessment (7 days, 10-point VAS)**	
Parameter	Mean ± SD
Postoperative pain	2.17 ± 3.49
Perceived swelling	1.33 ± 2.16
Difficulty chewing	1.67 ± 2.58
Difficulty speaking	1.67 ± 2.58
Interference with daily activities	1.33 ± 2.16
Need for analgesics	3.50 ± 4.37
Overall surgical experience	9.67 ± 0.82
**Final satisfaction assessment (1–2 months, 5-point Likert scale)**	
**Parameter**	Mean ± SD
Surgery more comfortable than expected	4.50 ± 0.84
Robotic technology provided a sense of safety	5.00 ± 0.00
Surgery perceived as highly precise	5.00 ± 0.00
Procedure less invasive than expected	4.67 ± 0.82
Recovery was rapid	4.83 ± 0.41
Would choose robotic surgery again	5.00 ± 0.00
Would recommend treatment to others	5.00 ± 0.00
Overall satisfaction with treatment	5.00 ± 0.00

Abbreviations: VAS, visual analogue scale (0 = none; 10 = maximum); Likert scale (1 = strongly disagree; 5 = strongly agree).

## Data Availability

The data presented in this study are available from the corresponding author upon reasonable request.

## Supplementary Materials

The following supporting information can be downloaded at: https://www.mdpi.com/article/10.3390/dj14080528/s1, File S1: Patient-reported outcome measures (PROMs) questionnaire used to assess preoperative expectations, postoperative recovery, and final satisfaction following robotic-assisted full-arch implant rehabilitation.
