# Supplementary material for: Clinical Accuracy and Patient Experience Following Robotic-Assisted Full-Arch Implant Rehabilitation: A Case Series
Source: Dent J (Basel). 2026 Aug 18;14(8):528. doi: 10.3390/dj14080528 (PMC13512193; doi:10.3390/dj14080528)
Supplement: Supplementary file 1 [file dentistry-14-00528-s001.zip › dentistry-4427837-supplementary.pdf]

Questionnaire

Robotic-Assisted Dental Implant Surgery

Instructions: Please answer all questions honestly according to your personal experience. Your responses will remain confidential and will be used exclusively for scientific purposes.

PART A — PREOPERATIVE QUESTIONNAIRE

Visual Analog Scale (VAS): 0 = none / 10 = maximum

Question	Score
What level of anxiety do you feel before the surgery?	________
How much confidence does robotic-assisted surgery inspire in you?	________
To what extent do you believe robotic technology can improve surgical accuracy?	________
What expectations do you have regarding the treatment outcome?	________
How much fear do you have of postoperative pain?	________

PART B — EARLY POSTOPERATIVE QUESTIONNAIRE (24 HOURS AND 7 DAYS)

Visual Analog Scale (VAS): 0 = none / 10 = maximum

Question	Score
Postoperative pain	________
Perceived swelling	________
Difficulty chewing	________
Difficulty speaking	________
Interference with daily activities	________
Need for analgesic medication	________
Overall surgical experience	________

PART C — FINAL SATISFACTION QUESTIONNAIRE (1–3 MONTHS)

Likert Scale: 1 = strongly disagree / 5 = strongly agree

Question	Score
The surgery was more comfortable than I expected.	________
Robotic technology made me feel safe during the treatment.	________
I perceived the surgery as highly precise.	________
The procedure was less invasive than I expected.	________
My recovery was rapid.	________

I would choose robotic-assisted surgery again.	__________
I would recommend this treatment to other patients.	__________
Overall, I am satisfied with the treatment received.	__________
